# SpatialSort: a Bayesian model for clustering and cell population annotation of spatial proteomics data

**DOI:** 10.1093/bioinformatics/btad242

**Published:** 2023-06-30

**Authors:** Eric Lee, Kevin Chern, Michael Nissen, Xuehai Wang, IMAXT Consortium, Chris Huang, Anita K Gandhi, Alexandre Bouchard-Côté, Andrew P Weng, Andrew Roth

**Affiliations:** Department of Molecular Oncology, BC Cancer Agency, 675 West 10th Avenue, Vancouver, BC V5Z1L3, Canada; Graduate Bioinformatics Training Program, University of British Columbia, 100-570 West 7th Avenue, Vancouver, BC V5T4S6, Canada; Department of Statistics, University of British Columbia, 2207 Main Mall, Vancouver, BC V6T1Z4, Canada; Terry Fox Laboratory, British Columbia Cancer Research Centre, 675 West 10th Avenue, Vancouver, BC V5Z1L3, Canada; Terry Fox Laboratory, British Columbia Cancer Research Centre, 675 West 10th Avenue, Vancouver, BC V5Z1L3, Canada; CRUK IMAXT Grand Challenge Consortium, Li Ka Shing Centre, Robinson Way, Cambridge CB20RE, United Kingdom; Translational Medicine Hematology, Bristol Myers Squibb, 86 Morris Ave, Summit, NJ 07901, United States; Translational Medicine Hematology, Bristol Myers Squibb, 86 Morris Ave, Summit, NJ 07901, United States; Department of Statistics, University of British Columbia, 2207 Main Mall, Vancouver, BC V6T1Z4, Canada; Terry Fox Laboratory, British Columbia Cancer Research Centre, 675 West 10th Avenue, Vancouver, BC V5Z1L3, Canada; Department of Pathology and Laboratory Medicine, University of British Columbia, 2211 Wesbrook Mall, Vancouver, BC V6T1Z7, Canada; Department of Molecular Oncology, BC Cancer Agency, 675 West 10th Avenue, Vancouver, BC V5Z1L3, Canada; Department of Pathology and Laboratory Medicine, University of British Columbia, 2211 Wesbrook Mall, Vancouver, BC V6T1Z7, Canada; Department of Computer Science, University of British Columbia, 2366 Main Mall, Vancouver, BC V6T1Z4, Canada

## Abstract

**Motivation:**

Recent advances in spatial proteomics technologies have enabled the profiling of dozens of proteins in thousands of single cells in situ. This has created the opportunity to move beyond quantifying the composition of cell types in tissue, and instead probe the spatial relationships between cells. However, most current methods for clustering data from these assays only consider the expression values of cells and ignore the spatial context. Furthermore, existing approaches do not account for prior information about the expected cell populations in a sample.

**Results:**

To address these shortcomings, we developed SpatialSort, a spatially aware Bayesian clustering approach that allows for the incorporation of prior biological knowledge. Our method is able to account for the affinities of cells of different types to neighbour in space, and by incorporating prior information about expected cell populations, it is able to simultaneously improve clustering accuracy and perform automated annotation of clusters. Using synthetic and real data, we show that by using spatial and prior information SpatialSort improves clustering accuracy. We also demonstrate how SpatialSort can perform label transfer between spatial and nonspatial modalities through the analysis of a real world diffuse large B-cell lymphoma dataset.

**Availability and implementation:**

Source code is available on Github at: https://github.com/Roth-Lab/SpatialSort.

## 1 Introduction

Recently developed high-throughput technologies for spatial protein expression profiling can perform highly multiplexed phenotyping of single cells, while preserving the spatial organization of tissues. Examples of these technologies include imaging mass cytometry (IMC) (Giesen et al. 2014), multiplexed ion beam imaging (MIBI) (Angelo et al. 2014), and codetection by indexing imaging (CODEX) (Goltsev et al. 2018). These technologies have the capacity to quantify dozens of protein markers at single-cell resolution *in situ*, providing an opportunity to enhance studies of cellular heterogeneity by going beyond the quantification of cellular composition and allowing for direct inference of cell-to-cell interactions from spatial context.

A key step in the analysis of spatial data is assigning cells to their constituent cellular populations as defined by expression profiles (e.g. T-cells, B-cells, malignant cells, etc.) (Azizi et al. 2018; Keren et al. 2018; Schürch et al. 2020; Jackson et al. 2020; Ali et al. 2020; Rendeiro et al. 2021). The dominant paradigm for performing this analysis is to cluster cells based on their expression profile and then perform *post hoc* annotation of the clusters based on known markers that delineate cell types. We demonstrate that such a procedure is suboptimal and that new approaches tailored to spatial expression data are required.

The clustering step of most two-step analyses have been performed using methods developed for disaggregated single cell data (Azizi et al. 2018; Keren et al. 2018; Schürch et al. 2020; Jackson et al. 2020; Ali et al. 2020; Rendeiro et al. 2021), such as PhenoGraph (Levine et al. 2015) and Scanpy (Wolf et al. 2018). A limitation of disaggregate methods is that they ignore spatial information, in particular the identity of neighbouring cells. Neighbourhood information can be highly informative when inferring the cell types, for example if cell types tend to associate due to receptor–ligand signalling. Most methods that account for spatial interactions of expression data have been developed for spatial transcriptomics (Zhu et al. 2018; Zhao et al. 2021; Yang et al. 2022) with the Giotto package (Dries et al. 2021) adapting the method from (Zhu et al. 2018) for proteomics data. Like our proposed method, these tools are based on Hidden Markov Random Field (HMRF) models (Kindermann 1980; Bishop 2006), and as many perform dimensionality reduction of the data which is then modelled by a Normal distribution, they could be applied to spatial proteomic data. However, approaches such as BayesSpace (Zhao et al. 2021) and SC-MEB (Yang et al. 2022) assume the neighbourhood structure defining the HMRF graph is fixed to match spot based spatial transcriptomics platforms. Furthermore, these approaches only model automonomous cell–cell interactions, assuming the parameter controlling the strength of this interactions is either fixed such as in (Zhao et al. 2021) or learned via grid search as in (Zhu et al. 2018; Dries et al. 2021; Yang et al. 2022) This autonomous cell type interaction assumption amounts to “smoothing” the assignment of cells in close proximity to originate from the same population. This assumption fails to capture more complex biological scenarios involving nonautonomous signalling between cells of different types. For example, malignant Reed–Sternberg cells are known to extensively associate with non-malignant cells within the tumour microenvironemnt of Hodgkin lymphomas (Scott and Gascoyne 2014). Our first contribution in this work is to develop a generalized HMRF model capable of handling nonautonomous neighbour interactions, where the parameter controlling the strength of these interactions is jointly learned with cluster assignments.

The annotation of clusters to identify their cell type in two-step procedures is typically performed manually. This is problematic as it can be subjective and difficult to reproduce (Zhang et al. 2019). Additionally, separating the annotation step from clustering means that valuable “prior” information about the expression profiles expected for each cluster are ignored, requiring methods to learn de novo the expression profiles of clusters. While a significant number of methods have been developed to address the cell type annotation problem for disaggregated single cell data (Pasquini et al. 2021), we are not aware of any approaches that incorporate spatial information. Thus, our second contribution in this work is to provide several options for performing joint spatially aware clustering and cell type annotation, which is not currently supported by any of the existing methods. As we show in the results, this approach improves clustering accuracy while negating the need to perform laborious and subjective manual cluster annotation.

To address these issues outlined above we have developed a Bayesian model, SpatialSort, to jointly perform spatially aware clustering and cell type annotation. The input is a cell-by-marker expression profile matrix and a graph representing the spatial adjacencies between pairs of cells. To capture spatial dependencies between cells, SpatialSort models cell labels using an HMRF. The model accounts for different cell types' propensities to be neighbours through an interaction matrix whose entries indicate the affinity of cell types to neighbour each other. We fit the model using Markov Chain Monte Carlo (MCMC) methods, and the output is a clustering of cells with optional annotation of cell type identity for each cluster. To test the performance of SpatialSort, we conducted benchmarking experiments using synthetic and semireal datasets. Furthermore, we applied SpatialSort to a real-world diffuse large B-cell lymphoma (DLBCL) dataset profiled with MIBI. Our results demonstrate that SpatialSort is able to leverage spatial information and prior knowledge of cell type composition to improve the clustering and annotation of spatial expression data.

## 2 Methods

### 2.1 Probabilistic spatially aware clustering with SpatialSort

**Figure 1.**
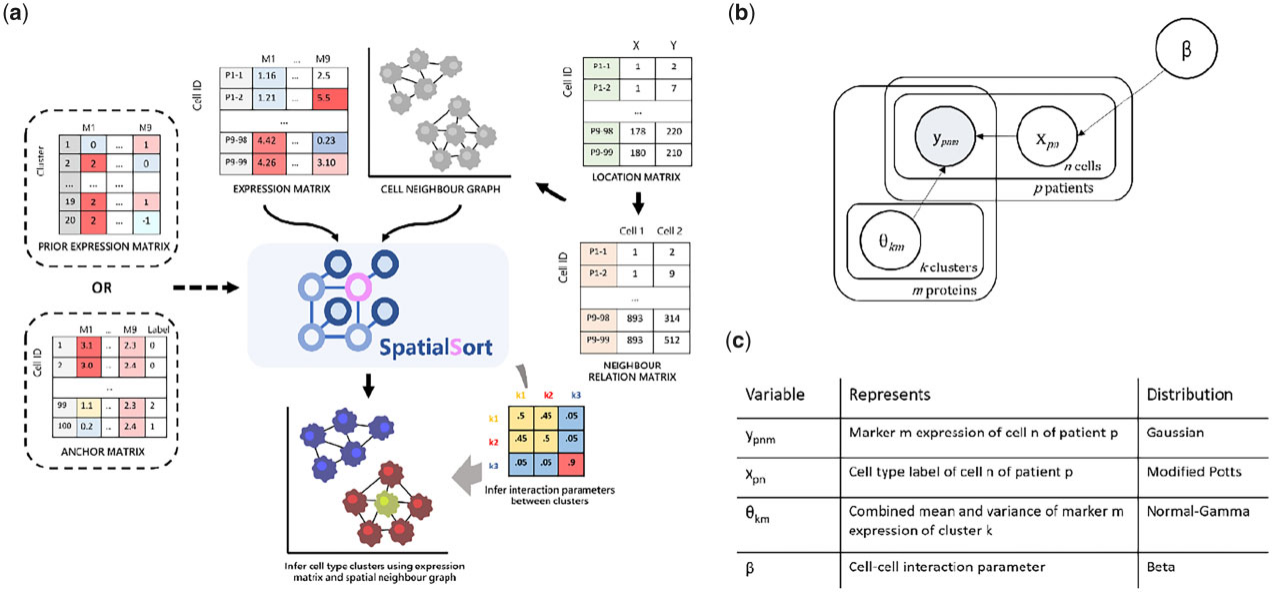
(a) Schematic overview, (b) probabilistic graphical model, and (c) prior distributions of SpatialSort. SpatialSort requires expression, cell location, and neighbour relation data as inputs. For each patient, a neighbour graph modelled by a MRF is built to represent the spatial context. Using both expression and spatial data for inference, SpatialSort jointly infers cluster assignment and the interaction parameter of the HMRF to probabilistically assign each cell to a given cell type cluster. When an expectation of certain cell types or a collection of labelled data is present, a prior expression matrix or an anchor expression matrix can be incorporated to improve clustering or perform label transfer.

We provide a detailed overview of the SpatialSort model and inference procedure here along with a schematic diagram in Fig. 1a. SpatialSort jointly considers cell expression values and neighbourhood spatial structure to perform clustering. To perform unsupervised clustering, SpatialSort requires inputs consisting of a multisample marker by cell expression matrix and a list of sample-specific cell location matrices from spatial expression profiling, which is used to identify neighbour cells. Neighbouring cells are defined as cells having a spatial proximity less than a user set threshold in pixels. SpatialSort takes the cell location and neighbour relations to construct sample-specific cell connectivity graphs that link cells that are spatially proximal. To capture the non-random spatial associations between cell types, SpatialSort uses a HMRF to allow cells to influence the cluster assignments of their neighbours (Fig. 1b and c). As exact Bayesian inference for HMRF models is intractable, SpatialSort uses MCMC sampling to approximate the posterior distribution and estimate model parameters.

SpatialSort can be used in a completely unsupervised way when no prior information about cell populations is available. However, in most spatial proteomic studies, markers are chosen to discriminate between known cell populations. SpatialSort provides two modes to incorporate information regarding these known populations' expression profiles: Prior mode and Anchor mode.

Prior mode takes an additional input of a population by marker matrix, which encodes knowledge of the degree of expression per marker in each cell population. Anchor mode involves the introduction of anchor cells, which are expression profiles of cells measured by previous assays and assigned to cell populations. Multiple cells from each population may be included in the set of anchors, which can better reflect the variability of expression within the population and help SpatialSort infer the expected variance of marker expressions. Anchor cells may be measured using either disaggregated or spatial technologies, though the major constraint is that a reasonable number of overlapping markers should be covered in both the anchor and query datasets and the anchor dataset should be suitably transformed to have expression which match the query dataset.

Both prior and anchor modes can be used to discover unknown populations. In prior mode, clusters can be specified with vague priors for all markers. In anchor mode, clusters can be specified with no anchor cells.

### 2.2 A generative model for spatially-aware clustering of expression data

SpatialSort is an instance of a HMRF model. HMRFs models are defined on an undirected graph $G=\left(E,V\right),\;\;$where E is the set of edges in the graph and V are the set of (labelled) nodes. Because the graph is undirected, we assume that E is a set of sets, where elements of E are sets of the form {u,v} with u,v∈ V.

Let the observed data be denoted by ${Y=\{y_{n}\}}_{n=1}^{N}$, where N is the total number of cells and N=|V|. A data point $y_{n}\in \;R^{M}\;$is the measured expression profile of a cell, where M denotes the number of proteins measured. Each cell n has an associate latent variable, $x_{n}\in \{1,\;\ldots ,\;K\}$, indicating its cell population (cluster) assignment. K is the number of clusters. The collection of cell labels ${X=\{x_{n}\}}_{n=1}^{N}$, then form the node labels of V defining the Markov Random Field (MRF) graph. The MRF assumptions means that the value of $x_{n}$ depends on the values of its immediate neighbours in the graph. We denote the set of neighbours of n∈ V by N(n) = {n | {n, n′}} ∈ E. The strength of influence neighbouring cells has on the label of each other is governed by K×K affinity matrix which we denote by β. The specification of the priors for the entries of β is deferred to the next section where we describe variants of the SpatialSort model.

Each cluster k has an associated parameter $\theta_{k}$, which represents the mean and precision of expression of proteins for cells associated with cluster k. Each component of $\theta_{k}$, denoted $\theta_{km}$, is assumed to be independent and given a NormalGamma prior distribution. Given $x_{n}$ and ${\theta =\{\theta_{k}\}}_{k=1}^{K}$ we assume the values of $y_{n}$ are conditionally independent. The full joint distribution for the model is given in Equation (1).


(1) $$p\left(X,\;Y,\;\theta ,\;\beta \;\right)=p\left(\beta \right)\;p\left(X|\beta \right)\;p\left(\theta \right)\;\prod_{n=1}^{N}p\left(y_{n}\;\right|{\;x}_{n},\;\theta )\;$$

The term $p\left(X\right|\beta )\;$describes the MRF component of the joint distribution. The MRF distribution is a product of terms for each edge in the graph. Each term in the product is the exponential of the entry in the matrix β corresponding to the identity of contributing edges. The unnormalized form of $p\left(X\right|\beta )\;$is given in Equation (2).


(2) $$p\left(X\;|\;\beta \;\right)=\exp(\sum_{\left\{n,\;n^{'}\right\}\in E}\beta_{{\;x}_{n},\;{\;x}_{n^{'}}})$$

The normalization constant Z(β) of $p\left(X\right|\beta )\;$can be found by summing over all possible values of ${X=\{x_{n}\}}_{n=1}^{N}$, which is intractable for all but small values of *N*. As we discuss later this poses an inferential challenge when updating β.

The full hierarchical model, except for the specification of β, is as follows:


$$\theta_{km}=\left(\mu_{km},\;\tau_{km}\right)\;∼\;\mathrm{NormalGamma}\left(\cdot \right|\mu_{0},\;\lambda_{0},\;\alpha_{0},\;\beta_{0})$$

$$X\;|\;\beta \;∼\;\mathrm{MRF}\;\left(\cdot \right|\beta )$$

$$y_{nm}|x_{nm}=k,\;{\left\{\theta_{l}\right\}}_{l=1}^{K}∼\;\mathrm{Normal}\left(\cdot \right|\mu_{km},\;\tau_{km})$$

The model can be trivially extended to multiple samples or regions of interest by treating each new sample as separate connected components of the MRF graph.

### 2.3 Specifying the affinity matrix

Because the MRF graph is undirected, the affinity matrix β is assumed to be symmetric, thus there are up to $K(K+1)/2\;\in \;O(K^{2})$ free parameters that need to be specified. In practice, it is neither computationally feasible nor statistically efficient to treat all entries of β as free parameters. Here we discuss several parameterisations of β which lead to different variants of the SpatialSort model.

The simplest and most commonly employed parameterisations of β is to use a single value, $\beta^{s}$, which is shared across all diagonal entries and setting the off diagonals to 0, i.e. *β*_*kk*_$=\beta^{s}$and *β_kl_* =0 for k≠ l (Zhu et al. 2018; Zhao et al. 2021; Dries et al. 2021; Yang et al. 2022). This simple model, often referred to as the Potts model, captures affinities of cells of the same type and assumes that they all have the same strength. Due to the intractability of the normalization constant Z(β) of $p\left(X\right|\beta ),\;$it is common to fix $\beta^{s}$ (Zhao et al. 2021). We refer to the variants of SpatialSort with $\beta^{s}$ fixed as the 0p and with $\beta^{s}$ estimated as the 1p model. For the 1p model, we assign $\beta^{s}$ a Uniform(0,1) prior. For the 0p model, we fix $\beta^{s}$ to 0.5 for all analyses performed in this work.

The limitation of the standard Potts model is the inability to capture affinities between clusters (cell populations) of different types. To address this, we consider a richer parameterization of β which allows for variable strengths of autonomous interactions and allows for nonautonomous interactions. We refer to this model as the *K*p model, as there are K parameters which need to be estimated. In the *K*p model the diagonals of β are set to *β_kk_* = *β_k_^s^* which accounts for variable affinities for autonomous interactions. We define *β_k_^d^* = 1 − *β_k_^s^* and let *β_kl_* = (*β_k_^d^*+*β_l_^d^*)/2 for the off diagonal terms to capture nonautonomous interactions. We assign a Uniform(0,1) prior to *β_k_^s^*.

### 2.4 Incorporating prior knowledge into clustering

Incorporating prior knowledge of marker proteins to define clusters can improve clustering accuracy. To support this, a quaternary coded K×M prior expression matrix can be provided as an additional input parameter to SpatialSort. Each row of the matrix represents a prior belief about the expression levels of markers for a cluster. Values from 0 to 2 are used to code the mean parameter $\mu_{km}$ of $\theta_{km}$ into the 25th, 50th, and 75th percentiles of expression for each marker expression of *Y*. The value -1 is used when no prior knowledge is available and associated with a mean of zero and a high variance.

Another way to incorporate prior knowledge is to directly leverage previously annotated cell types and anchor clusters to specific expression profiles. The introduced cells are referred to as “anchors,” as they influence the updates of cell cluster assignments and strongly anchor clusters to a specific expression signature profile. Anchors have a fixed cluster assignment and do not contribute to the HMRF graph. The anchors act to specify the distribution parameters of their associated clusters, thus improving the accuracy of clustering and allowing for label transfer between disaggregate and spatial datasets.

### 2.5 Inference of latent cluster labels and cell–cell interactions

To infer X and β, we must compute the marginalized posterior distribution of X and β, given the data Y (Equation 3).


(3) $$P\left(X,\;\beta \;|\;Y\;\right)\propto P\left(Y\right|\beta ,\;X)\;P\left(X\right|\beta )\;P\left(\beta \;\right)$$

Exact computation of the posterior distribution is intractable, so we instead use MCMC sampling methods to approximate it. Cell labels $x_{n}$ are sampled through a collapsed Gibbs sampler. The interaction parameters β are sampled via a Double Metropolis-Hastings (DMH) sampler (Liang 2010). One full iteration of the inference algorithm performs five updates of β using the DMH algorithm and one update of X using Gibbs sampling.

### 2.6 Obtaining point estimates of the Markov Chain Monte Carlo trace

For all experiments on synthetic and real datasets, we ran SpatialSort for 500 iterations. The first half of the MCMC trace was removed as burn-in.

To derive a point estimate for X from the MCMC trace we use two different strategies depending on whether prior information is supplied. When no prior information is provided the cluster labelling is unidentifiable. Thus, we use the Maximisation of Posterior Expected Adjusted Rand (MPEAR) criterion to summarize the post-burn-in trace (Fritsch and Ickstadt 2009). In prior and anchor mode clusters become identifiable, and thus we use the last sampled value of X in the trace.

### 2.7 Preprocessing

For the semireal dataset experiments, a 13-dimensional CyTOF dataset of bone marrow mononuclear cells were downloaded from (Levine et al. 2015). Cells without labels from gating were discarded. An arcsinh transformation was applied to normalize the dataset. Dimensional reduction with principle component analysis (PCA) was performed on the markers for unsupervised clustering, anchor mode, and Gaussian mixture model (GMM).

For the real-world DLBCL dataset experiments, CyTOF DLBCL datasets were normalized by marker against a spike-in control to account for machine drift and batch effects in staining. This dataset was then normalized by a hyperbolic arcsinh function. MIBI DLBCL datasets were also normalized by a hyperbolic arcsinh function and divided by 10 to reduce expression intensity to the same scale as CyTOF. As there were no common B cell lineage marker between CyTOF and MIBI, CD19 and PAX5 were treated as equivalent. In the anchor experiments, spatially aware downsampling through breadth first search was performed on the MIBI data to 2000 cells per sample. Additional subsetting was done on both CyTOF and MIBI datasets to retain only overlapping markers: CD45, CD19/PAX5, CD3, CD4, CD8, CD45RO, CD57, CXCR5, and PD-1. Dimensional reduction with PCA was performed on the common cell type lineage markers between the two modalities. The top six principal components were used as input for label transferring.

### 2.8 Alternative methods

We used a GMM as implemented in the scikit-learn package version 0.24.2 (Pedregosa 2011). For comparing anchors and priors, we ran SpatialSort with a disconnected neighbour graph, to mimic nonspatial prior/anchor informed clustering, henceforth referred to as non-SpatialSort. The number of components for the GMM and non-SpatialSort was the same used for SpatialSort. Popular methods Phenograph v1.5.7 (Levine et al. 2015), Scanpy (Leiden clustering) v1.9.2 (Wolf et al. 2018), and Giotto (HMRF) v3.2.0 (Dries et al. 2021) with default parameters were also tested. Clustering accuracy was assessed using the V-Measure metric (Rosenberg and Hirschberg 2007).

## 3 Results

### 3.1 Modelling nonautonomous cell interactions increases accuracy

We first sought to explore the impact of incorporating spatial information during clustering. To do so, we simulated data from the SpatialSort model with nonautonomous cell-to-cell affinities. To simulate real spatial structure, we applied breadth-first search on neighbourhood graphs generated from a previous IMC study (Ali et al. 2020) to maintain the spatial structure of the subset graph. We explored variations of expression values and spatial structure by generating 100 datasets each for two types of HMRF interactions parameters, which we refer to as “biased” and “uniform.” Biased refers to the condition where cells of the same cluster had a stronger affinity to be grouped together spatially, whereas uniform referred to the case where affinities were sampled from a uniform distribution. We used these datasets to evaluate the three variants of the SpatialSort model that differ in the number of parameters used to model cell-to-cell affinities (see Section 2). We also include a GMM in the comparison, as a nonspatial baseline to calibrate the performance of the different SpatialSort variants.

**Figure 2.**
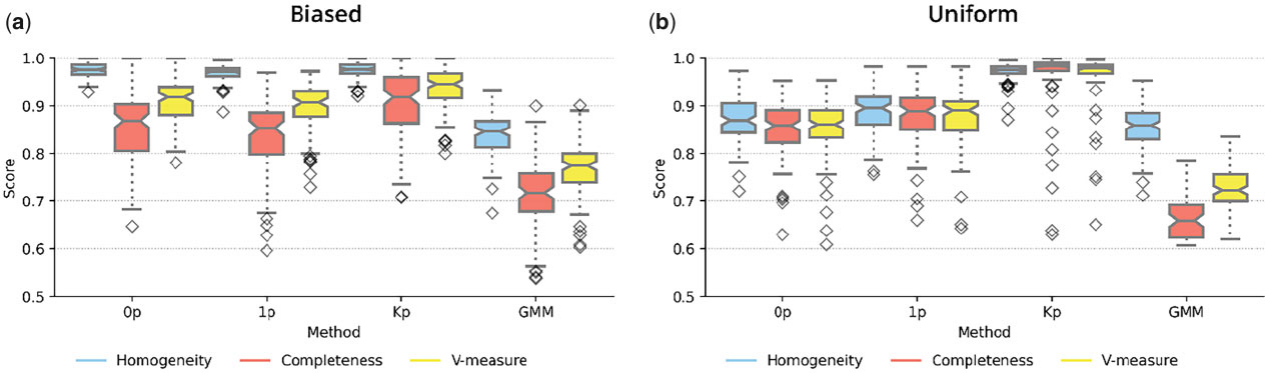
Comparison of performance on model fitting on forward simulated datasets for (a) biased and (b) uniform datasets. Methods applied are shown on the *x*-axis. 0p indicates the Potts model, 1p and *K*p are different parameterisations of the Potts model, GMM indicates the Gaussian mixture model. Performance is shown on the *y*-axis scored by metrics of homogeneity, completeness, and V-measure which are colour coded according to the legend.

The results are summarized in Fig. 2 for the biased and uniform datasets, respectively. Clustering accuracy was assessed using the V-Measure metric, with a value of 1.0 indicating perfect accuracy (Rosenberg and Hirschberg 2007). When comparing methods, we applied the Friedman test to see whether there were any significant differences in performance between the methods (*P*-value <.01). If the Friedman test was significant, we then applied the *post hoc* Nemenyi test with a Bonferroni correction to all pairs of methods to determine which methods showed significantly different performance from each other (*P*-value <.01) (Demšar 2006). All statements of significance are with respect to this procedure.

All variants of the SpatialSort model significantly outperformed the nonspatial GMM baseline in our experiments. When comparing the spatial models, the *K*p model had significantly better accuracy than both the 0p and 1p models for both biased and uniform datasets. For the biased dataset, the V-measure was on average 0.027 and 0.057 higher for the *K*p model when compared with the 0p and 1p models, respectively. The performance delta between *K*p and simpler spatial models was much larger for the uniform datasets. The *K*p model had an average increase of V-measure of 0.112 and 0.093 over the 0p and 1p models, respectively.

The increased delta in performance between the *K*p and simpler spatial models supports the notion that explicitly accounting for nonautonomous cell to cell interactions can lead to significant gains in performance when such interactions are present.

### 3.2 SpatialSort is robust to overlapping expression profiles

We posited that accounting for spatial structure would improve cluster assignment in the case of cells with similar expression profiles. To explore this hypothesis, we simulated data using the same strategy as the previous synthetic experiment, but varied the degree of overlap in marker expression distributions. Marker expressions were modelled using Gaussian mixtures that were generated using the MixSim R package (Melnykov et al. 2012), which allowed for controllable overlap of simulate expression profiles. We evaluated across five different overlaps from 0.025 to 0.125 and varied spatial structure by generating 50 datasets for each overlap under both biased and uniform interaction parameters. For this analysis we consider only the *K*p variant of the SpatialSort model, henceforth referred to as SpatialSort. We compared against GMM as a baseline, and Phenograph (Levine et al. 2015) which is a widely used clustering approach for spatial data. We again applied the Friedman and *post hoc* Nemenyi test to assess statistical significance.

**Figure 3.**
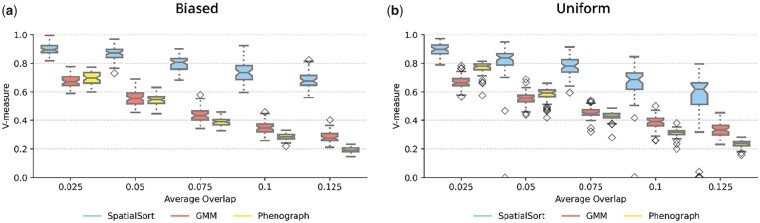
Comparison of performance on model fitting on spatial Gaussian mixture datasets for (a) biased and (b) uniform datasets. The average overlaps of spatial Gaussian mixture datasets are shown on the *x*-axis. Scores of performances using the V-measure metric are shown on the *y*-axis. Each dataset with different average overlap was fit by three different methods: SpatialSort, GMM, and Phenograph.

The results from this experiment are summarized in Fig. 3. SpatialSort significantly outperformed the GMM and Phenograph for all overlap values on both the biased and uniform datasets. The average increase of V-measure for SpatialSort versus GMM ranged from 0.210 to 0.399 and versus Phenograph ranged from 0.128 to 0.492. The performance of all methods degraded as the degree of overlap in expression profiles increased. However, SpatialSort's performance was significantly more robust to increasing overlap. This trend held for both biased and uniform datasets. These results support the hypothesis that spatial information can help to more accurately cluster cell types with similar expression profiles.

### 3.3 Prior information improves accuracy

We next sought to explore the impact of incorporating prior information during clustering. To do so we generated *semi-real* datasets by using real cell expression profiles from a 13-dimensional CyTOF bone marrow mononuclear cell data. Cell labels for this dataset were obtained by manual gating in a previous study (Bendall et al. 2011) and used as ground truth for our analysis. Cell neighbourhood graphs and node labels were generated the same way as the synthetic experiments. Expression values were associated with nodes in the graph by assigning a cell from the corresponding cluster in the CyTOF data. We explored variations of clusters and spatial structure by generating 100 datasets for the biased and uniform interaction parameters. The compositions of cell types was similar when simulating data with either of the two types of HMRF interaction parameter settings with the difference in datasets manifesting in the spatial organization of cells. We ran SpatialSort in unsupervised, prior and anchor modes. For comparison, we tested: GMM, Phenograph, Scanpy and Giotto HMRF. To quantify the relative contribution of spatial versus prior/anchor information, we also ran non-SpatialSort as described in the methods. We performed PCA to reduce the dimensionality of the data to eight dimensions for GMM, Giotto, unsupervised (non-)SpatialSort and (non-)SpatialSort with anchors. No dimensionality reduction was applied when using prior mode for (non-)SpatialSort, as specifying prior values of principle components was not a realistic use case. Phenograph was also run without PCA dimensionality reduction, as it applies its own dimensionality reduction. Scanpy Leiden clustering was run with default parameters except we used 100 neighbours.

**Figure 4.**
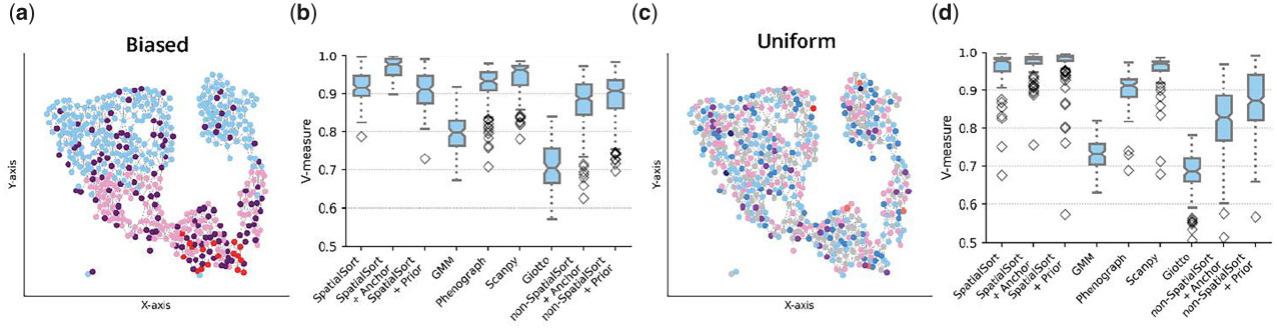
Performance on semireal spatial CyTOF data. (a) A spatial neighbour graph of a singular sample in the biased dataset. Nodes indicate a single cell colour-coded by cluster assignment. Cells tend to engage in autonomous interactions spatially. (b) Boxplot of V-measure scores to show clustering accuracy of various methods fitting on 100 semireal biased datasets. (c) and (d) are examples of the uniform dataset as a comparison. Uniform interaction terms render cells to have a random chance of neighbouring any type of cell.

The results of this experiment are summarized in Fig. 4b and d. Scanpy performed the best out the nonspatial methods. Surprisingly, Giotto HMRF performed uniformly worse than all other methods. This may be a result of suboptimal tuning obtained by following the Giotto tutorial. When using prior mode, Scanpy was significantly better than SpatialSort on the biased data and significantly worse on the uniform data. SpatialSort demonstrated its best performance in the anchor mode. Using anchors SpatialSort significantly outperformed all other methods both the biased dataset and the uniform dataset. SpatialSort in unsupervised mode was outperformed in all cases by both prior and anchor modes. SpatialSort with anchors significantly outperformed the non-SpatialSort on both datasets, whereas SpatialSort with priors only significantly outperformed its nonspatial equivalent on the uniform datatset. These results suggest the including prior or anchors information significantly improves the accuracy of spatially aware clustering. Furthermore, ablation testing with non-SpatialSort indicates the benefits of prior information is additive with the incorporation of spatial information.

### 3.4 Employing anchors to characterize the spatial architecture of DLBCL

To illustrate the real-world utility of SpatialSort we next analysed a MIBI dataset of 116 000 cells from a cohort of 29 patients with DLBCL. For each patient, two regions of interest (ROI) were obtained to address variations in tumour content. We also incorporated the expression data of 128 673 cells from a previously clustered CyTOF assay of the same 29 patients to provide anchors for the characterization of the cellular composition of the tumour microenvironment in the MIBI data. Data preprocessing is described in the methods. We then ran the 0p, 1p, and *K*p models with anchors to perform label transferring.

**Figure 5.**
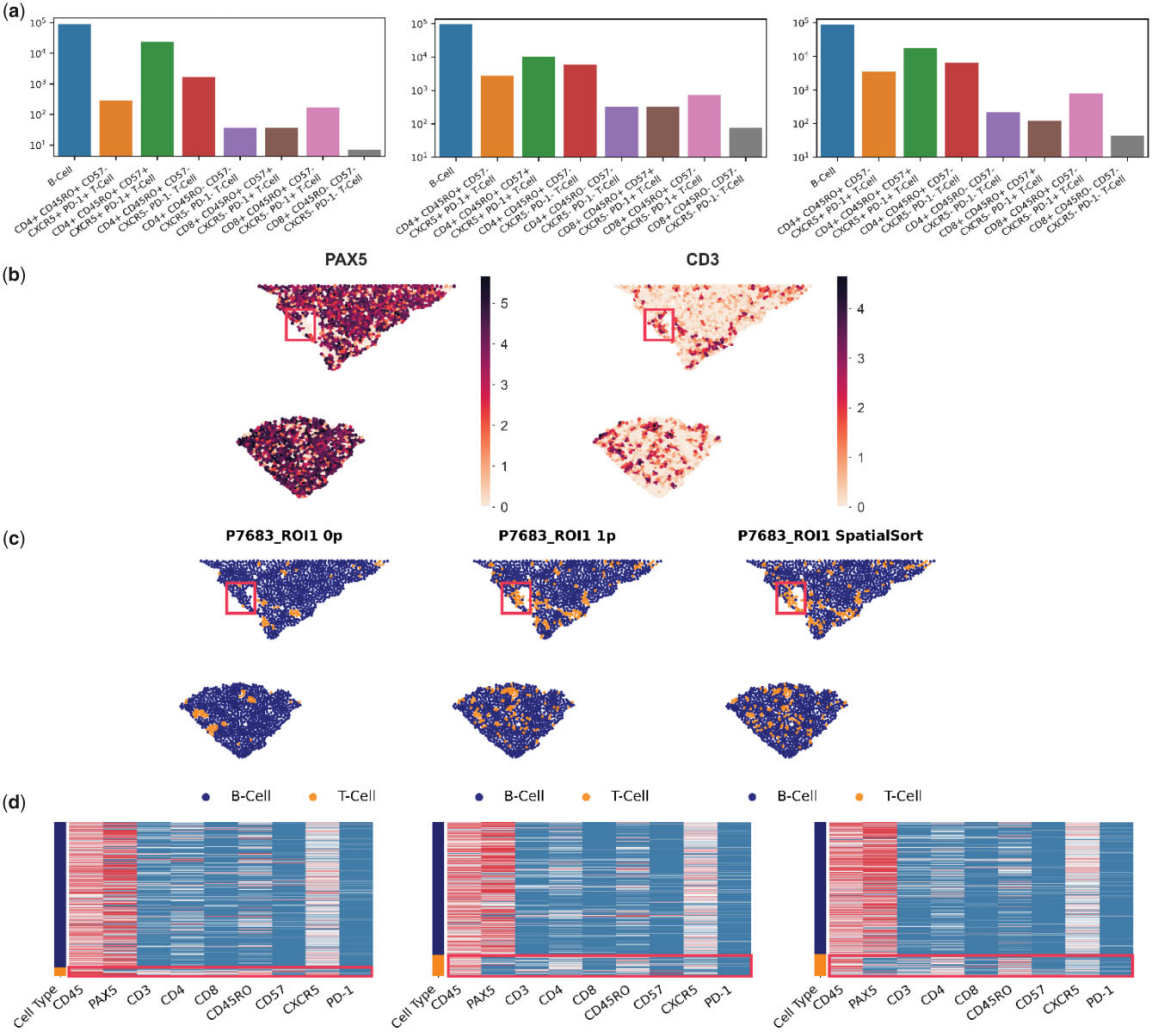
Cell type annotation of DLBCL MIBI data using SpatialSort. (a) The cell type distribution bar graph of the clustering results from using the 0p, 1p, and *K*p model. Counts are log-scaled. (b) Spatial distribution of the expression of lymphocyte lineage markers, PAX5 and CD3 in sample P7683. Colour represents normalized intensity of expression. (c) Sample P7683 plotted by spatial coordinates. Cells are colour-coded by cell type assignment inferred by the 0p, 1p, and *K*p models in anchor mode. The red box in b and c highlights one area of significant difference in cell assignment. (d) Sample-specific expression heatmaps for sample P7683. Rows are colour-coded by cell type shown in (c). T-cells are highlighted by red boxes to illustrate mixed PAX5 expression.

The results of this analysis are summarized in Fig. 5. With spatial data, we were able to investigate the interaction matrices which indicate the observed frequency of two cell types to be spatially proximal. All three spatial models were able to capture the strong autonomous interaction between B cells, with 71%, 52%, and 53% of edges being B-cell autonomous in the 0p, 1p, and *K*p models, respectively. However, we observed a significant difference (*P*-value <.001, Pearson chi-squared test) in the cell type distribution estimated by the 0p model compared with the 1p and *K*p models (Fig. 5a).

Visualisation with cluster specific heatmaps revealed some clusters from the 0p model having higher disparity in expression patterns between cells than that of 1p and *K*p models. The Davies-Bouldin score (Davies and Bouldin 1979), of the 1p and *K*p models were superior at 1.92 compared with the 0p model at 2.67, with a lower score indicating higher coherence and less noise within clusters. Additionally, visualization of the cellular associations in the spatial structure using patient-specific neighbourhood graphs depicted an oversmoothing effect from the 0p model compared with the 1p and SpatialSort models (Fig. 5b–d). An exemplar from sample P7683 illustrates that the *K*p model can more effectively resolve cell types consistent with lineage marker intensities (B-cell PAX5; T-cell CD3) and effectively distinguish between cell types with overlapping expression profiles. Furthermore, these results suggest that the non-random associations between cellular phenotypes in the spatial structure can be more effectively identified when autonomous and nonautonomous interactions are inferred in spatially aware clustering.

## 4 Discussion

SpatialSort provides two important advancements over current state of the art methods for analysing spatial protein expression data. First, SpatialSort accounts for potential affinities between nonautonomous cell neighbours while clustering. This more accurately models the underlying biology and improves over the smoothing approach implicit in current HMRF based models (Zhu et al. 2018; Zhao et al. 2021; Yang et al. 2022). Second, SpatialSort provides the ability to incorporate prior information about the expected cellular populations present. This improves upon *post hoc* labelling of clusters due to the fact that prior information is directly incorporated while clustering, increasing accuracy.

SpatialSort’s main limitation is computational complexity due to the challenges of posterior inference. The posterior distribution is doubly intractable because not only is the normalization constant of the posterior distribution difficult to evaluate explicitly, as is typical for Bayesian models, but also the likelihood of the HMRF. Previous HMRF based approaches have avoided this issue by using a single autonomous affinity value set manually (Zhu et al. 2018; Zhao et al. 2021; Yang et al. 2022), thus avoiding the need to compute the normalization constant of the HMRF. Our results suggest this limits current HMRF methods to effectively be spatial smoothers. We address this issue using the DMH algorithm to approximately sample from the posterior. However, this precludes the possibility of using more computationally efficient approaches such as expectation maximization and variational methods for inference. Despite this, our analysis of real datasets with over 100 000 cells took on average 1.1 min per sampling iteration or 9 h to perform an entire run on a personal laptop computer. For extremely large datasets we would suggest downsampling the number of cells based on a breadth first search of the neighbour graphs. We note this may induce bias, and would recommend users explore multiple downsamplings from different seeds. Though we have not explored it in this work, there is also significant opportunity for parallelization across disconnected components of the neighbour graph.

One caveat to highlight about the inferred affinity matrices β is that they are likely not interpretable parameters. In calibration experiments (not shown) simulating from full $O(K^{2})$ affinity matrices, we have found that the *K*p model could not reliably recover the true affinity values. This is likely due to the constrained form of the affinity matrix in the *K*p model, which we enforce for computational efficiency. Despite this, we still observed that the *K*p model improved performance relative to the 0p and 1p models in the presence of nonautonomous effects, which we further validate with the results shown. Thus, while the *K*p model provides the ability to model nonautonomous interactions to improve labelling performance, we advise against using the inferred values as the basis for statistical comparison.

The neighbour graph of cells will certainly impact the results of our method. While it is beyond the scope of this work, exploration of different distance thresholds for defining neighbour relations is recommended for users. Recent work has demonstrated the importance of such multiscale spatial analysis (Roemer et al. 2022).

In this work we have primarily focused on the application of SpatialSort to proteomic data. However, there is no reason the model could not be modified to work with transcriptomic data. Following existing transcriptomic approaches (Zhao et al. 2021; Yang et al. 2022), dimensionality reduction with PCA could be applied. An alternative approach we leave for future work would be to replace the Normal emission distribution for the data with discrete distribution such as a Negative-Binomial (Love et al. 2014).

We believe SpatialSort will be a valuable contribution to the spatial expression toolbox for many biologists. It addresses several unmet needs in the field and identifies several novel issues that have thus far been ignored.

## Data Availability

Raw data for all the experiments used in this article have been deposited in Zenodo with DOI: https://doi.org/10.5281/zenodo.6909419.
